# Transverse Electric Inverse Scattering of Buried Conductors in a Slab Medium Using DSM and U-Net

**DOI:** 10.3390/s26154942

**Published:** 2026-08-04

**Authors:** Chien-Ching Chiu, Po-Hsiang Chen, Yen-Chen Chang, Eng Hock Lim

**Affiliations:** 1 Department of Electrical and Computer Engineering, Tamkang University, Tamsui 251301, Taiwan; phchen@mail.tku.edu.tw (P.-H.C.); 411490120@o365.tku.edu.tw (Y.-C.C.); 2 Department of Electrical and Electronic, University Tunku Abdul Rahman, Kajang 43200, Malaysia; limeh@utar.edu.my

**Keywords:** inverse scattering problem, buried conductor, electromagnetic imaging, transverse electric (TE), direct sampling method (DSM), U-Net, slab medium

## Abstract

This paper presents a DSM-initialized U-Net framework for reconstructing the shape of a perfectly conducting target buried in a slab medium from measured electromagnetic scattered fields. The conductive target is irradiated with Transverse Electric (TE) waves, followed by the acquisition of the resulting scattered fields. These scattered field data were first used for forward scattering calculations with the Method of Moments (MoM), followed by preliminary shape reconstruction using the DSM. The preliminary images received from the DSM were then input into a U-Net for imaging. Compared with the free space case, the slab medium condition limited the incident and receiving angles. Numerical results indicate that the standalone DSM provides only an approximate estimate of the conductor profile, whereas the proposed DSM–U-Net framework achieves average NRMSE values of 6.90% under 5% Gaussian noise and 8.95% when trained with 5% noise and tested with 10% noise.

## 1. Introduction

Electromagnetic imaging, a vital emerging technology utilized across biomedicine, remote sensing, and medical imaging, operates by emitting waves to illuminate an unknown scattering object and subsequently retrieving its properties size, profile, material, and position from the received scattered field. However, electromagnetic imaging remains persistently challenged by the intrinsic nonlinearity and ill-posedness of the Inverse Scattering Problem (ISP), and traditional solutions that convert the ISP into a time-consuming optimization problem with high computational cost have critically failed to achieve high-resolution and real-time imaging.

At present, strategies for addressing electromagnetic inverse problems have primarily branched into objective function methods [1,2,3,4,5,6] and neural network-based learning methods [7,8,9,10,11,12,13,14,15,16]. Objective-function-based strategies are commonly adopted to locate the minimum or maximum of a target expression—for example, by reducing the discrepancy between measured data and the simulated scattered fields. Considerable progress has been made in this field over the past twenty years. Electromagnetic imaging techniques developed under this framework can generally be grouped into two major categories: local exploration and global exploration schemes. Customarily, the search for an optimal solution involves either local gradient-descent techniques or broad-scale heuristic search algorithms. The former is more effective when the search region is confined, whereas the latter is preferable when broader parameter spaces must be explored.

In research on objective function methods applied to microwave imaging, in 2021, Wei proposed the Finite-Difference Frequency-Domain (FDFD), the conventional iterative approach, and the distorted FDFD-based iterative scheme. Such techniques circumvent the analytical challenges associated with deriving Green’s functions for irregularly configured environments. It is demonstrated numerically that the proposed methods deliver accurate and high-fidelity reconstruction results [1]. In 2022, Xie proposed the dominant current coefficient method to solve inverse scattering problems at multiple frequencies. Numerical results indicated that the proposed method reconstructs the region of interest under low-frequency conditions and subsequently utilizes high-frequency signals to reconstruct the image of the object [2]. In 2023, Wu proposed an effective scattering-center model based on Inverse Synthetic Aperture Radar (ISAR) imagery. The model characterized the properties of target scattering centers. By integrating scattering-center theory, it enabled the reconstruction of target images under arbitrary poses. Numerical experiments, compared with the linear interpolation, confirmed the effectiveness of the proposed approach [3]. In 2023, Dubey proposed the Distorted wave extended Phaseless Rytov Iterative Method (DxPRIM) to address phaseless data inverse scattering problems. Built upon an extended Rytov Rpproximation (RA), the method substantially expanded the applicability of conventional RA-based formulations. Both simulation and experimental results demonstrated that DxPRIM could successfully reconstruct scatterers using only phase-free measurement data [4]. In 2024, Üregen introduced a novel algorithm that integrated physical linearization techniques with Newton’s method. This approach alleviated the nonlinear and ill-posed nature of the inverse problem while removing the reliance on an accurate initial guess by generating it iteratively within the algorithm. Numerical results indicated that, compared with the traditional multi-incidence Newton method, the proposed scheme achieves a substantially lower reconstruction error and reduced computation time by nearly a factor of three [5]. In 2025, Zhou proposed an adaptive damping mechanism and integrated it into the Variational Born Iterative Method (VBIM) framework to enhance the solution efficiency and stability of ISPs. Experimental results demonstrated that this method, when applied to both synthetic and measured data, not only achieved faster convergence but also maintained good inversion accuracy [6].

Recently, a range of deep learning methods have been developed to strengthen the performance of microwave imaging. In 2021, Guo proposed a novel Generative Adversarial Network (GAN) to enhance the resolution of preliminary estimated images [7]. In 2022, Liu proposed the Physical Model-Inspired Deep Unrolling Network (PM-Net), which aims to combine traditional model-based methods with deep learning strategies to solve non-linear electromagnetic inverse scattering problems. Numerical results indicated that, compared to existing deep learning methods, PM-Net requires fewer training parameters and achieves superior overall performance. This demonstrates the high potential of the approach in integrating physical priors with data-driven models [8]. In 2023, Liu proposed a physics-informed unsupervised deep learning framework, together with a cost-effective strategy for adaptive data acquisition using information metasurfaces. Instead of relying on labeled datasets, the method leveraged physical laws to guide the data-acquisition process. Experimental results demonstrated that this approach significantly accelerated data acquisition while preserving high reconstruction quality [9]. In 2024, Wu proposed a physics-induced deep learning architecture. In this framework, a contrastive learning network provided initial contrast source estimates, and an attention-based semantic segmentation model was applied to suppress the strong nonlinear effects of multiple scattering. Numerical results demonstrated that this approach significantly enhances the reconstruction accuracy of the dielectric constant [10]. In 2025, Maricar proposed a U-Net architecture that incorporated attention gates to improve reconstruction accuracy. Numerical experiments demonstrate that the proposed method achieves superior reconstruction accuracy and higher computational efficiency compared with existing baseline techniques [11]. In 2025, Liu showed Convolution Neural Network (CNN)-Mamba2-Net for 3-Dimensional Electromagnetic Tomography (3D EMT) image reconstruction. The CNN branch captured fine-grained local features, while the Mamba-2 branch, equipped with a linear attention mechanism, effectively modeled the 3D spatial structure of the input data. Experimental results demonstrated that this hybrid design enabled more accurate feature extraction and representation, achieving strong performance in 3D EMT reconstruction tasks [12].

Previous TE-wave inverse scattering studies have commonly formulated the reconstruction problem as an iterative optimization process, which requires repeated forward solutions and consequently entails considerable computational cost. Compared with TM-wave inversion, TE-wave reconstruction is further complicated by the coupling between transverse electric-field components, because the tangential electric-field boundary condition involves spatial derivatives of the longitudinal magnetic field. This coupling increases the sensitivity of the reconstruction to target geometry and measurement noise. Moreover, multiple reflections and refractions in the slab medium, together with the restricted transmitter and receiver locations in Regions 1 and 3, reduce the available angular information and further aggravate the ill-posedness of the inverse problem [13,14]. These limitations motivate the proposed DSM–U-Net framework, in which DSM provides a computationally efficient initial estimate of the target location, while U-Net refines the incomplete boundary information contained in the DSM reconstruction.

To address these limitations, the proposed framework combines DSM with U-Net. DSM provides a computationally efficient initial estimate of the target location without requiring iterative forward-model updates, whereas U-Net refines the incomplete and distorted boundary information contained in the DSM image. This combination is intended to improve conductor-shape reconstruction under limited-view TE-wave measurements in a slab medium.

Motivated by these challenges, non-iterative approaches—such as the DSM—have gained increasing interest owing to their low computational burden and strong tolerance to measure noise. DSM forms an indicator directly from the observed scattered data, enabling fast identification of the target region without relying on iterative algorithms or any prior knowledge [15,16,17]. However, the DSM methods cannot recover the detail of the dielectric constant of the objects. Distinguishing itself from prior literature, this study offers several major advancements, outlined below:

1. While significant advancements for inverse problems of conductors have been achieved, prior studies have centered almost exclusively on TM waves, leaving the potential of TE wave imaging under-explored. Compared with previous studies that mainly focus on TM-wave or free-space configurations, this work investigates a DSM–U-Net framework for the TE-wave reconstruction of perfectly conducting targets buried in a slab medium.
2. Because the slab medium configuration permits measurements solely in the upper and lower domains, the resulting reconstruction process is more demanding than that in free-space environments. However, using DSM combined with U-Net can yield good results.
3. The nonlinear nature of TE waves exacerbates defects. The results obtained from numerical simulation experiments verify that the suggested method attains strong reconstruction capability and refined image quality for conductor profiles in TE-wave-based imaging.

In this work, the inverse problem of perfect conductors buried in a planar medium under TE irradiation is tackled using U-Net with initial shape estimation by DSM. Given the inherently strong nonlinear characteristics of TE wave scattering, the integration of neural networks is crucial for improving image sharpness and reconstruction accuracy. The remainder of this paper is structured as follows: Section 2 outlines the theoretical principles of subsurface electromagnetic scattering and describes the application of the DSM for preliminary imaging. Section 3 elaborates on the CNN architecture used for the reconstruction task. Section 4 provides a comprehensive analysis of the numerical simulation results to validate the effectiveness of the proposed method. Lastly, Section 5 concludes the paper by synthesizing the principal research findings and discusses potential directions for future research.

## 2. Theoretical Models

**Figure 1:**
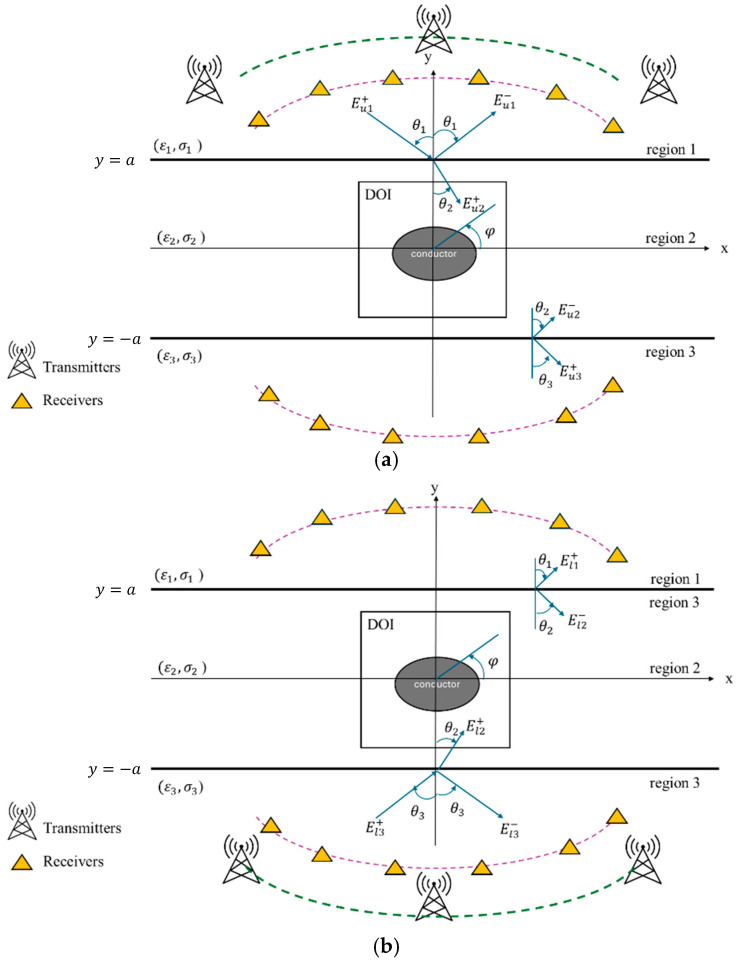
Unknown conductor in a slab medium: (**a**) Region 1 incident; (**b**) Region 3 incident.

The theoretical foundation governing the scattering of TE wave waves is delineated in this section, providing the basis for the subsequent inversion algorithm. A perfect conductor is assumed to be buried within a slab medium and extends infinitely along the z-axis. As illustrated in Figure 1, the transverse profile within the xy-plane is given by the polar representation ρ = F(θ). The excitation source is formulated as a harmonic in time, TE polarized plane wave.

In the three-layer space, we divide the incident method into an upper incident and lower incident. The upper half-incident is presented in Figure 1a, and the lower incident is presented in Figure 1b. The electric field incident from region 1 is given by Equation (1). $\theta_{1}$ is the incident angle. $\theta_{2}$ and $\theta_{3}$ are the refraction angles.

(1) $${\bar{E}}_{u}^{i}\left(x,y\right)=\left\{\begin{array}{c} \eta_{1}H_{u1}^{+}\left\{\left[\overset{\wedge }{x}cos\theta_{1}+\overset{\wedge }{y}sin\theta_{1}\right]e^{-jk_{1}\left[cos\theta_{1}\left(-y\right)+sin\theta_{1}x\right]}\right\}\\ +\eta_{1}H_{u1}^{-}\left\{\left[-\overset{\wedge }{x}cos\theta_{1}+\overset{\wedge }{y}sin\theta_{1}\right]e^{-jk_{1}\left[cos\theta_{1}\left(y\right)+sin\theta_{1}x\right]}\right\},y\geq a\\ \eta_{2}H_{u2}^{+}\left\{\left[\overset{\wedge }{x}cos\theta_{2}+\overset{\wedge }{y}sin\theta_{2}\right]e^{-jk_{2}\left[cos\theta_{2}\left(-y\right)+sin\theta_{2}x\right]}\right\}\\ +\eta_{2}H_{u2}^{-}\left\{\left[-\overset{\wedge }{x}cos\theta_{2}+\overset{\wedge }{y}sin\theta_{2}\right]e^{-jk_{2}\left[cos\theta_{2}\left(y\right)+sin\theta_{2}x\right]}\right\},a>y>-a\\ \eta_{3}H_{u3}^{+}\left\{\left[\overset{\wedge }{x}cos\theta_{3}+\overset{\wedge }{y}sin\theta_{3}\right]e^{-jk_{3}\left[cos\theta_{3}\left(-y\right)+sin\theta_{3}x\right]}\right\},y\leq -a \end{array}\right.$$

Here, $H_{u1}^{+}$ represents the incident magnetic field in Region 1, taken to be 1. $H_{u2}^{+}$ and $H_{u3}^{+}$ denote the transmission coefficients in Region 2 and Region 3. $H_{u1}^{-}$ and $H_{u2}^{-}$ indicate the reflection parameters in Region 1 and Region 2, respectively.$\eta_{1}$, $\eta_{2}$, and $\eta_{3}$ are the medium’s characteristic impedance. The electric field incident from region 3 is given by Equation (2). $\theta_{3}$ is the incident angle. $\theta_{2}$ and $\theta_{1}$ are the refraction angles.

(2) $${\bar{E}}_{l}^{i}(x,y)=\left\{\begin{array}{c} \eta_{1}H_{l3}^{+}\left\{\left[-\overset{\wedge }{x}cos\theta_{3}+\overset{\wedge }{y}sin\theta_{3}\right]e^{-jk_{3}\left[cos\theta_{3}\left(-y\right)+sin\theta_{3}x\right]}\right\}\\ +\eta_{1}H_{l3}^{-}\left\{\left[\overset{\wedge }{x}cos\theta_{3}+\overset{\wedge }{y}sin\theta_{3}\right]e^{-jk_{3}\left[cos\theta_{3}\left(y\right)+sin\theta_{3}x\right]}\right\},y\geq a\\ \eta_{2}H_{l2}^{+}\left\{\left[-\overset{\wedge }{x}cos\theta_{2}+\overset{\wedge }{y}sin\theta_{2}\right]e^{-jk_{2}\left[cos\theta_{2}\left(-y\right)+sin\theta_{2}x\right]}\right\}\\ +\eta_{2}H_{l2}^{-}\left\{\left[\overset{\wedge }{x}cos\theta_{2}+\overset{\wedge }{y}sin\theta_{2}\right]e^{-jk_{2}\left[cos\theta_{2}\left(y\right)+sin\theta_{2}x\right]}\right\},a>y>-a\\ \eta_{3}H_{l1}^{+}\left\{\left[-\overset{\wedge }{x}cos\theta_{1}+\overset{\wedge }{y}sin\theta_{1}\right]e^{-jk_{1}\left[cos\theta_{1}\left(-y\right)+sin\theta_{1}x\right]}\right\},y\leq -a \end{array}\right.$$

Here, $H_{l3}^{+}$ represents the incident magnetic field in Region 3, taken to be 1. $H_{l2}^{+}$ and $H_{l1}^{+}$ represent the transmission coefficients in Region 2 and Region 1. $H_{l3}^{-}$ and $H_{l2}^{-}$ indicate the reflection parameters in Region 3 and Region 2, respectively.

By virtue of the electromagnetic equivalence principle, the scattered magnetic field, defined as ${\overset{⃑}{H}}^{s}=\overset{⃑}{H}-{\overset{⃑}{H}}^{i}$, at an arbitrary observation point represented by point (x, y) in the Cartesian system or (r, θ) in polar coordinates can be expressed as follows:

(3) $${\overset{⃑}{H}}^{s}\left(x,y\right)=\int_{c}G(x,y;x\prime ,y\prime )\left(j\omega \varepsilon \right)J_{sm}\left(\bar{r}\prime \right){dl}^{\prime }$$

where c denotes the outer contour of the conductor. $J_{sm}(\bar{r}\prime )$ denotes current density at the conductor’s surface. r represents (x, y). $G\left(\overset{⃑}{r},{\overset{⃑}{r}}^{\prime }\right)$ is the Green’s function.

(4) $$G\left(\overset{⃑}{r},{\overset{⃑}{r}}^{\prime }\right)=G\left(x,y;x^{\prime },y^{\prime }\right)=\left\{\begin{array}{c} G_{1}\left(x,y;x^{\prime },y^{\prime }\right),\;y\geq a\\ G_{2}\left(x,y;x^{\prime },y^{\prime }\right),a>y>-a\\ G_{3}\left(x,y;x^{\prime },y^{\prime }\right),y\leq -a \end{array}\right.$$

(5) $$G_{1}\left(x,y;x^{\prime },y^{\prime }\right)=\frac{1}{2\pi }\int_{-\infty }^{\infty }je^{-j\gamma_{1}\left(y-a\right)}\frac{\left(\gamma_{2}+\gamma_{3}\right)e^{j\gamma_{2}(y^{\prime }+a)}+\left(\gamma_{2}-\gamma_{3}\right)e^{-j\gamma_{2}(y^{\prime }+a)}}{\left(\gamma_{1}+\gamma_{2}\right)\left(\gamma_{2}+\gamma_{3}\right)e^{j\gamma_{2}(2a)}+\left(\gamma_{1}-\gamma_{2}\right)\left(\gamma_{2}-\gamma_{3}\right)e^{-j\gamma_{2}(2a)}}e^{-j\alpha (x-x^{\prime })}d\alpha$$

(6) $$\begin{array}{c} G_{2}\left(x,y;x^{\prime },y^{\prime }\right)=\frac{1}{2\pi }\int_{-\infty }^{\infty }\frac{j}{2\gamma_{2}}\left[\frac{\left(\gamma_{2}+\gamma_{1}\right)\left(\gamma_{2}+\gamma_{3}\right)e^{-j\gamma_{2}\left[\left|y-y^{\prime }\right|-2a\right]}+\left(\gamma_{2}-\gamma_{1}\right)\left(\gamma_{2}-\gamma_{3}\right)e^{j\gamma_{2}\left[\left|y-y^{\prime }\right|-2a\right]}}{\left(\gamma_{2}+\gamma_{1}\right)\left(\gamma_{2}+\gamma_{3}\right)e^{j\gamma_{2}\left(2a\right)}+\left(\gamma_{1}-\gamma_{2}\right)\left(\gamma_{2}-\gamma_{3}\right)e^{-j\gamma_{2}\left(2a\right)}}\right.\\ \;\left.+\frac{(\gamma_{2}-\gamma_{1})\left(\gamma_{2}+\gamma_{3}\right)e^{j\gamma_{2}\left[y+y^{\prime }\right]}+(\gamma_{2}-\gamma_{3})\left(\gamma_{2}+\gamma_{1}\right)e^{-j\gamma_{2}[y+y\prime ]}}{\left(\gamma_{2}+\gamma_{1}\right)\left(\gamma_{2}+\gamma_{3}\right)e^{j\gamma_{2}(2a)}+\left(\gamma_{1}-\gamma_{2}\right)\left(\gamma_{2}-\gamma_{3}\right)e^{-j\gamma_{2}(2a)}}\right]e^{-j\alpha (x-x^{\prime })}d\alpha \end{array}$$

(7) $$G_{3}\left(x,y;x^{\prime },y^{\prime }\right)=\frac{1}{2\pi }\int_{-\infty }^{\infty }je^{j\gamma_{3}\left(y+a\right)}\frac{\left(\gamma_{2}+\gamma_{1}\right)e^{-j\gamma_{2}(y^{\prime }-a)}+\left(\gamma_{2}-\gamma_{1}\right)e^{j\gamma_{2}(y^{\prime }-a)}}{\left(\gamma_{2}+\gamma_{1}\right)\left(\gamma_{2}+\gamma_{3}\right)e^{j\gamma_{2}(2a)}+\left(\gamma_{1}-\gamma_{2}\right)\left(\gamma_{2}-\gamma_{3}\right)e^{-j\gamma_{2}(2a)}}e^{-j\alpha (x-x^{\prime })}d\alpha$$

Equations (5)–(7) represent the layered-medium Green’s functions for a source located in Region 2. Specifically, $G_{1}$ and $G_{3}$ describe the fields observed in Regions 1 and 3, respectively, whereas $G_{2}$ describes the field when both the source and observation points are located in Region 2. These Green’s functions account for the transmission, reflection, and propagation effects introduced by the slab interfaces. They are used in the MoM forward solver and are selected in the DSM formulation according to the region containing the receiver. $G_{1}\left(x,y;x^{\prime },y^{\prime }\right)$, $G_{2}\left(x,y;x^{\prime },y^{\prime }\right)$, and $G_{3}\left(x,y;x^{\prime },y^{\prime }\right)$ are the Green’s functions. Imposing the tangential boundary condition ensures that the total electric field is 0 on the conductor, resulting in the following integral expression for $J_{sm}\left(\bar{r}\right)$.

(8) $$\hat{n}\times \left(\frac{1}{j\omega \varepsilon_{2}}\nabla \times \overset{⃑}{H}\right)=0$$

(9) $$\hat{n}=\frac{\hat{x}\left(F\left(\theta \right)\cos\theta +F^{\prime }\left(\theta \right)\sin\theta \right)+\hat{y}\left(F\left(\theta \right)\sin\theta -F^{\prime }\left(\theta \right)\cos\theta \right)}{\sqrt{F^{2}\left(\theta \right)2+{F^{\prime }}^{2}\left(\theta \right)}}$$

where $\hat{n}$ denotes the outward-pointing unit normal vector orthogonal to the surface of the conductor. The direct scattering framework involves computing the scattering response $E^{s}$ based on a predefined object configuration. This is achieved by initially computing $J_{sm}$ from Equation (8). Equation (10) is formulated by manipulating the integral expression in Equation (8).

(10) $$-\hat{n}\times {\overset{⃑}{E}}^{i}=\hat{n}\times {\overset{⃑}{E}}^{s}$$

In accordance with the MoM, the conductive boundary is discretized into N infinitesimal segments, where each element’s induced magnetic current is approximated as a constant pulse.

(11) $$J_{sm}\left(\bar{r}\right)=\sum_{n=1}^{N_{d}}{\left(J_{sm}\right)}_{n}{\left(P_{c}\right)}_{n}$$

(12) $${\left(P_{c}\right)}_{n}=\left\{\begin{array}{c} 1,on△C_{n}\;\\ 0,otherwise \end{array}\right.$$

Here, the pulse function ${\left(P_{c}\right)}_{n}$ corresponds the basis function in the expansion, and $△C_{n}$ corresponds to the i^th^ arc segment of the object from $\theta =2\pi (i\;-\;1)/N_{d}$ to $\theta \;=\;2\pi /N_{d}$. $N_{d}$ represents the number of conductor segments.

(13) $$-\hat{n}\times {\overset{⃑}{E}}^{i}=[\frac{\left(F\left(\theta \right)\sin\theta -F^{\prime }\left(\theta \right)\cos\theta \right)}{\sqrt{F^{2}\left(\theta \right)+{F^{\prime }}^{2}\left(\theta \right)}}E_{x}^{i}+\frac{\left(F\left(\theta \right)\cos\theta +F^{\prime }\left(\theta \right)\sin\theta \right)}{\sqrt{F^{2}\left(\theta \right)+{F^{\prime }}^{2}\left(\theta \right)}}E_{y}^{i}]\hat{z}$$

(14) $$\hat{n}\times {\overset{⃑}{E}}^{\;s}=[\frac{\left(F\left(\theta \right)\cos\theta +F^{\prime }\left(\theta \right)\sin\theta \right)}{\sqrt{F^{2}\left(\theta \right)+{F\prime }^{2}\left(\theta \right)}}\sum_{n=1}^{N}{\left(G_{4}\right)}_{mn}{\left(J_{sm}\right)}_{n}+\frac{\left(F\left(\theta \right)\sin\theta -F^{\prime }\left(\theta \right)\cos\theta \right)}{\sqrt{F^{2}\left(\theta \right)+{F\prime }^{2}\left(\theta \right)}}\sum_{n=1}^{N}{\left(G_{5}\right)}_{mn}{\left(J_{sm}\right)}_{n}]\hat{z}$$

(15) $$G_{4}\left(x,y;x^{\prime },y^{\prime }\right)=\frac{\partial }{\partial x}G_{2}\left(x,y;x^{\prime },y^{\prime }\right)$$

(16) $$G_{5}\left(x,y;x^{\prime },y^{\prime }\right)=\frac{\partial }{\partial y}G_{2}\left(x,y;x^{\prime },y^{\prime }\right)$$

Here, ${\overset{⃑}{E}}^{\;s}$ represents the scattered electric field.

The DSM is a computationally efficient and direct reconstruction imaging scheme designed to reconstruct the location in space and geometric profile of unknown scatterers. Albeit initially developed for two-dimensional dielectric inverse problems under fixed plane-wave excitation, the technique has subsequently been adapted for more sophisticated imaging tasks. Its high computational efficiency comes from demanding avoiding procedures such as performing singular value decompositions or solving unstable integral equations. In addition, the DSM maintains good performance even when the data are corrupted by noise, making it a dependable tool for inverse scattering analysis. In this work, the DSM is particularly suitable for retrieving the boundaries and shapes of conducting objects, since conductors require only the recovery of their geometry and scale. The indicator employed in the DSM is given by

(17) $$\Psi \left(r_{q}\right)=\frac{1}{M_{i}}\sum_{l=1}^{M_{i}}\frac{\left|\langle {\overset{⃑}{H}}_{l}^{s}\left(r\right),G_{c}(r,r_{q})\rangle \right|}{\left\|{\overset{⃑}{H}}_{l}^{s}\left(r\right)\right\|\left\|G_{c}(r,r_{q})\right\|}$$

Here, $r_{q}$ corresponds a single sampling point, r means the receiver location with a total of $M_{r}$ receiver positions, and $G_{c}(r,r_{q})$, with c=1,3, denotes the Green’s function corresponding to receivers located in Region 1 or Region 3, respectively. $M_{i}$ represents the number of transmitters. ${\overset{⃑}{H}}_{l}^{s}\left(r\right)$ is the vector of $M_{r}\times 1$ and $G_{2}(r,r_{q})$ is the $M_{r}\times 1$ vector. If $\Psi \left(r_{q}\right)$≈1, then $r_{q}$ lies inside the unknown scattering object. And, if $\Psi \left(r_{q}\right)$≈0, $r_{q}$ is placed within the surrounding medium.

## 3. Neural Networks

**Figure 2:**
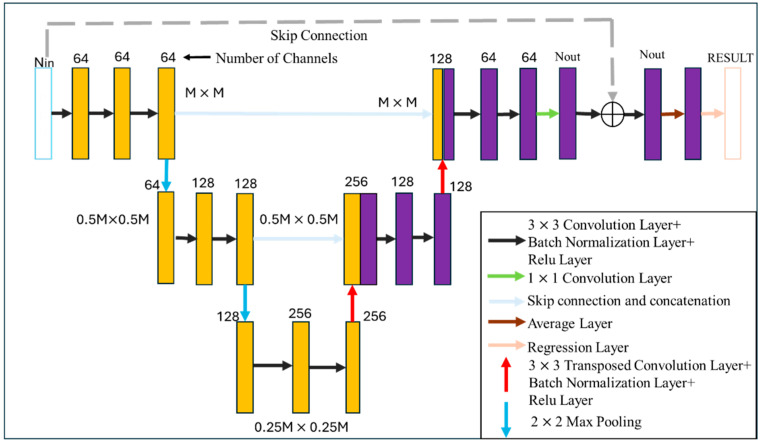
U-Net architecture.

For the accurate reconstruction of a perfectly conducting structure embedded in a slab medium, this study implements a specialized CNN based on the U-Net framework. As illustrated in Figure 2, the topological structure exhibits a symmetrical U-shaped design, bifurcated into a contracting path (encoder) on the left and an expanding path (decoder) on the right. This architecture is renowned for its data efficiency and modular flexibility, making it particularly advantageous for electromagnetic imaging tasks where training datasets are often limited.

The implemented U-Net takes a 64 × 64 DSM image as the input and produces a DSM reconstructed conductor image. The network consists of two encoder stages, one bottleneck stage, and two decoder stages. At the original spatial resolution of 64×64, three 3×3 convolutional layers with 64 filters are employed. Each convolutional layer uses the same padding and is followed by batch normalization and a ReLU activation function. A 2×2 max-pooling layer then reduces the feature-map resolution to 32×32. At this resolution, three convolutional layers with 64, 128, and 128 filters are applied, followed by a second 2×2 max-pooling layer.

At the bottleneck resolution of 16 × 16, three convolutional layers with 128, 256, and 256 filters are employed. In the decoder, 3 × 3 transposed convolutional layers are used to progressively restore the spatial resolution. At the 32×32 level, the upsampled feature maps are concatenated with the corresponding 128-channel encoder features, resulting in 256 channels, followed by two 3×3 convolutional layers with 128 filters. At the 64 × 64 level, the upsampled features are concatenated with the corresponding 64-channel encoder features, resulting in 128 channels, followed by two 3 × 3 convolutional layers with 64 filters.

A final 1×1 convolutional layer maps the extracted features to a single-channel output. In addition to the encoder–decoder skip connections, a global skip connection transfers the input DSM image to the output stage, where it is combined with the network prediction through element-wise addition, followed by an averaging layer and a regression layer [18]. Equation (18) represents the overall optimization objective used to determine the network parameters during training. The first term measures the discrepancy between the reconstructed conductor profile and the corresponding ground truth image, whereas Q(i) denotes the $L_{2}$ regularization term used to constrain the model and reduce overfitting. In this study, the reconstruction discrepancy is evaluated using the NRMSE loss defined in Equation (19). The term $Q\left(i\right)$ denotes the $L_{2}$-regularization applied to the trainable network weights, with a regularization coefficient of $1\times {10\;}^{-4}$. It penalizes excessively large weights to reduce overfitting. The minimization equation is formulated as

(18) argminAi,i:∑N=1NtfAi Fα, F+Qi

where $A_{i}$ denotes the neural network architecture, f refers to the error function, and $F^{\alpha }$ denotes the approximated conductor outline. The training procedure employs the Normalized Root Mean Square Error (NRMSE) as the loss function, defined as

(19) $$NRMSE=\frac{1}{N}\sum_{i=1}^{N}{\left\|T-T^{i}\right\|}_{F_{r}}/{\left\|T\right\|}_{F_{r}}$$

Here, T and $T^{i}$ represent the ground truth and the reconstructed image, respectively. N refers to the overall count of testing instances, and $F_{r}$ represents the Frobenius norm.

In the numerical results, the reconstruction performance of Deep Convolution Neural Network (DCNN) and U-Net was compared under the same experimental conditions. The DCNN directly maps the scattered-field data to the conductor profile, with the real and imaginary components of the scattered field arranged as a two-channel input. The network comprises three convolutional blocks followed by a fully connected output layer. The first and second convolutional layers employ 20 and 40 filters of the size 3×1, respectively, with a stride of 2×1. The third convolutional layer employs 80 filters of the size 2×2, with a stride of 1×1. All convolutional layers use the same padding, and each convolutional layer is followed by batch normalization and a ReLU activation function. The extracted features are subsequently mapped to the reconstructed conductor image through the fully connected layer [19]. To ensure a fair comparison, the DCNN and U-Net were trained using the same dataset partition and training hyperparameters.

## 4. Numerical Results

This research analyzes the image problem for conductors buried in a slab medium. To facilitate the inversion process, the conductive scatterers are excited by a 3 GHz TE polarized wave, thereby yielding the requisite scattered field measurements. The buried depth is chosen to be 0.2 m. Air serves as the background material in Regions 1 and 3 with the relative permittivity of $\varepsilon_{1}=\varepsilon_{3}=1$ and conductivity of $\sigma_{1}=\sigma_{3}=0$, whereas the background material in Region 2 is the wall with $\varepsilon_{2}=2.56$. 5% noise and 10% noise are added to the simulation. The transmitters are set up from −80° to 80° for $\theta_{1}$ or $\theta_{3}$ with 10° intervals in Region 1 or Region 3. The receivers are placed from $\varphi =20^{\circ }$ to 160° and φ=200° to 340° with 10° intervals in both Region 1 and Region 3, respectively. To summarize, the simulation involves 32 transmitters and 32 receivers. The computational domain, designated as the Domain of Interest (DOI), encompasses a spatial area of 0.16 m × 0.16 m, which is partitioned into a 64 × 64 discrete pixel grid. Conductive targets with different geometries were randomly positioned within the measurement domain, while the characteristic size of each target was about $\frac{\lambda }{2}$.

We leverage the Adaptive Moment (ADAM) optimizer for the learning process, owing to its widespread adoption and proven efficiency in handling large-scale neural network training. For the ADAM configuration, the learning rate is set within the range of $10^{-3}$ to $10^{-4}$ and is reduced by half every 20 epochs. The maximum number of epochs is 40, and the batch size is 32.

There are a total of 1512 (7 shapes × 9 positions × 6 orientations × 4 complementary-angle groups) samples. For each configuration, the scattered-field data obtained from 16 incident angles were divided into four complementary-angle groups, with each group containing four complementary illumination angles. The responses within each group were combined to compensate for the incomplete information obtained from a single illumination direction, resulting in four samples for each shape–position–orientation configuration. The dataset was randomly divided into 75% training samples and 25% testing samples. All forward-scattering simulations and inverse-scattering procedures, including the DSM reconstruction and neural network training and testing, were implemented in MATLAB R2025b. The Deep Learning Toolbox was used for the neural network implementation.

Additive Gaussian noise was introduced into the simulated scattered-field data before the DSM reconstruction. The noisy scattered-field vector is defined as

(20) $${\hat{E}}^{s}=E^{s}+n$$

where $E^{s}$ denotes the noise-free scattered-field vector, ${\hat{E}}^{s}$ denotes the noisy scattered-field vector, and n is an additive zero-mean Gaussian noise vector. For a prescribed noise level α, defined as the noise-to-signal power ratio, the corresponding signal-to-noise ratio is

(21) $$SNR_{dB}=10\log_{10}\left(\frac{1}{\alpha }\right)$$

The noise power is determined based on the average power of the simulated scattered-field vector as

(22) $$P_{n}=\alpha P_{s}$$

(23) $$P_{s}=\frac{1}{N}\sum_{m=1}^{N}{\left|E_{s}(m)\right|}^{2}$$

Here, $P_{n}$ and $P_{s}$ denote the noise power and the average scattered-field power, respectively, and N is the number of elements in the scattered-field vector. In this study, α=0.05 and 0.10, corresponding to SNR values of approximately 13.01 and 10 dB, respectively.

The shapes of the conductor shapes were generated using a Fourier-series representation:

(24) $$F\left(\theta \right)=a_{0}+\sum_{n=1}^{N/2}a_{n}\cos\left(n\theta \right)+\sum_{n=1}^{N/2}B_{n}\sin\left(n\theta \right)$$

In this paper, N=8. Here, F(θ) denotes the radial distance from the target center at the polar angle θ, $a_{0}$ specifies the nominal target size, and $a_{n}$ and $B_{n}$ denote the cosine and sine coefficients, respectively.

- Reconstruct the conductor shape

**Figure 3:**
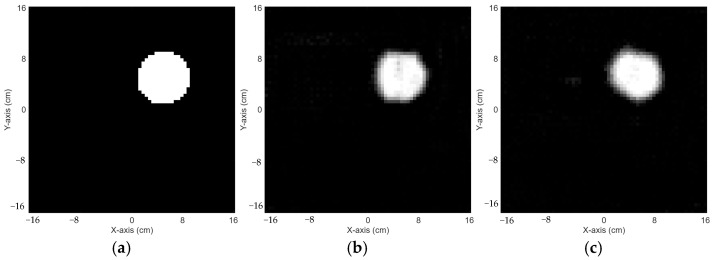
Imaging result of a circle shape: (**a**) Ground truth; (**b**) U-Net under 5% noise; (**c**) U-Net under training data with 5% noise and test data with 10% noise.

In case 1, a circle shape is used. Figure 3a presents the ground truth. Figure 3b shows its optional reconstructed image by U-Net with 5% noise. Figure 3c shows the image reconstructed by U-Net using training data with 5% noise and test data with 10% noise. Simulation results demonstrate that the proposed deep learning architecture significantly improves accuracy and continuity even in noisy conditions, enabling the precise reconstruction of conductor geometry. This comparative analysis validates the effectiveness of integrating neural network architectures to improve the fidelity of preliminary DSM results. This comparative analysis validates the efficacy of integrating neural network architectures to refine the fidelity of the preliminary results generated by the DSM. Table 1 lists the reconstruction accuracy and structure fidelity of seven different targets using U-Net under 5% noise. Table 2 lists the reconstruction accuracy and structure fidelity of seven different targets using U-Net under training data with 5% noise and test data with 10% noise. Both tables use NRMSE as evaluation metrics.

**Figure 4:**
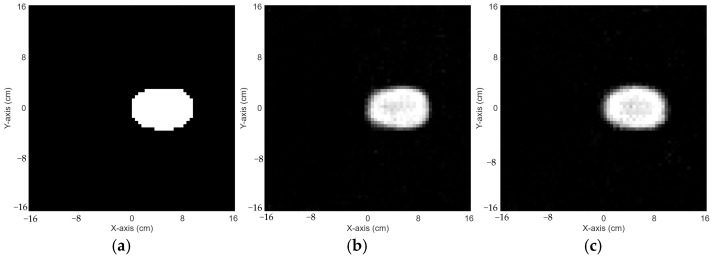
Imaging result of an oval shape: (**a**) Ground truth; (**b**) U-Net under 5% noise; (**c**) U-Net under training data with 5% noise and test data with 10% noise.

In case 2, an oval shape is used. Figure 4a presents the ground truth. Figure 4b shows its optional reconstructed image by U-Net under 5% noise. Figure 4c shows the image reconstructed by U-Net using training data with 5% noise and test data with 10% noise. The NRMSE value of the reconstructed image under 5% noise is 5.75%, as presented in Table 1. The NRMSE values of the reconstructed image under training data with 5% noise and test data with 10% noise are 6.29%, as shown in Table 2.

**Figure 5:**
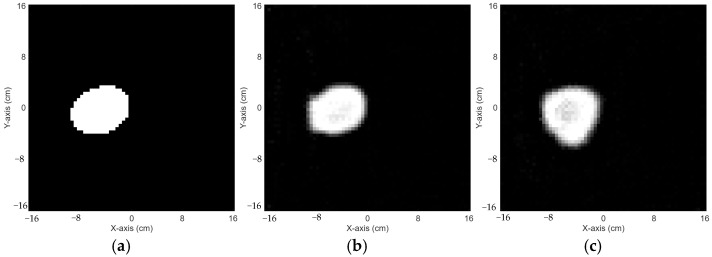
Imaging result of a pea shape: (**a**) Ground truth; (**b**) U-Net under 5% noise; (**c**) U-Net under training data with 5% noise and test data with 10% noise.

In case 3, a pea shape is used. Figure 5a presents the ground truth. Figure 5b shows its optional reconstructed image by U-Net under 5% noise. Figure 5c shows the image reconstructed by U-Net using training data with 5% noise and test data with 10% noise. Compared to the ground truth, the reconstruction of the lower portion of the figure is less satisfactory in this instance. The NRMSE values of the reconstructed image under 5% noise is 5.41%, as presented in Table 1. The NRMSE value of the reconstructed image under training data with 5% noise and test data with 10% noise is 9.61%, as presented in Table 2.

**Figure 6:**
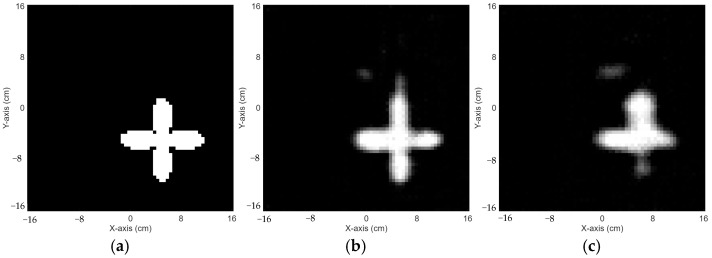
Imaging result of a cross shape: (**a**) Ground truth; (**b**) U-Net under 5% noise; (**c**) U-Net under training data with 5% noise and test data with 10% noise.

In case 4, a cross shape is used. Figure 6a presents the ground truth. Figure 6b shows its optional reconstructed image by U-Net under 5% noise. Figure 6c shows the image reconstructed by U-Net using training data with 5% noise and test data with 10% noise. Compared to the ground truth, the image in this reconstruction appears relatively blurred near the boundaries of the object. The NRMSE value of the reconstructed image under 5% noise is 8.46%, respectively, as presented in Table 1. The NRMSE value of the reconstructed image under training data with 5% noise and test data with 10% noise is 14.51%, as presented in Table 2.

**Figure 7:**
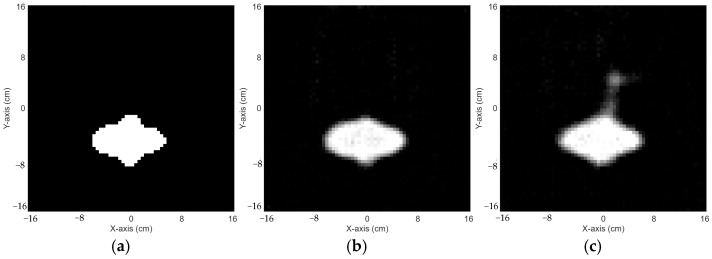
Imaging result of a UFO shape: (**a**) Ground truth; (**b**) U-Net under 5% noise; (**c**) U-Net under training data with 5% noise and test data with 10% noise.

In case 5, a UFO shape is used. Figure 7a presents the ground truth. Figure 7b shows its reconstructed image by U-Net under 5% noise. Figure 7c shows the image reconstructed by U-Net using training data with 5% noise and test data with 10% noise. Compared to the original shape, an additional shadow appears in the upper portion of the reconstructed figure. The NRMSE value of the reconstructed image under 5% noise is 7.33%, as presented in Table 1. The NRMSE value of the reconstructed image under training data with 5% noise and test data with 10% noise is 7.96%, as presented in Table 2.

**Figure 8:**
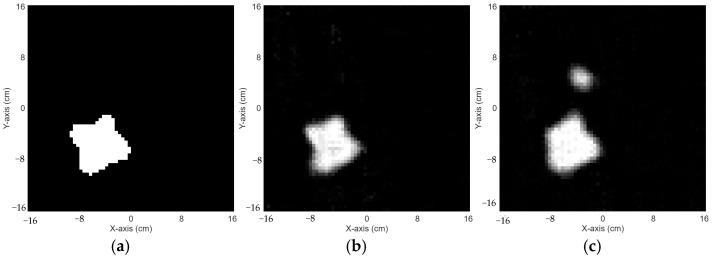
Imaging result of a four-petal shape: (**a**) Ground truth; (**b**) U-Net under 5% noise; (**c**) U-Net under training data with 5% noise and test data with 10% noise.

In case 6, a four-petal shape is used. Figure 8a presents the ground truth. Figure 8b shows its optional reconstructed image by U-Net under 5% noise. Figure 8c shows the image reconstructed by U-Net using training data with 5% noise and test data with 10% noise. Compared to the original shape, this reconstruction includes an additional shadow. The NRMSE value of the reconstructed image under 5% noise is 9.28%, as presented in Table 1. The NRMSE value of the reconstructed image under training data with 5% noise and test data with 10% noise is 9.58%, as presented in Table 2.

B. Large-Scale Dataset Validation and Comparative Reconstruction Evaluation

To further strengthen the experimental validation, the dataset was expanded to 28,392 samples, generated from 169 target positions, four complementary-angle groups, seven conductor shapes, and six data-augmentation variants. The DCNN and U-Net models were trained separately using the same data partitioning and training settings to ensure a fair comparison. The results show that U-Net consistently achieves better reconstruction performance than DCNN. In addition, independent models were trained and evaluated at 2 GHz and 3 GHz to investigate the influence of the operating frequency. The 3 GHz condition achieves better overall reconstruction accuracy than the 2 GHz condition, further supporting the selection of 3 GHz as the operating frequency in the preceding experiments. Despite the substantially enlarged dataset and increased positional diversity, the results demonstrate consistent reconstruction performance across the tested target shapes, spatial positions, and operating frequencies within the same MoM-based forward-simulation model.

**Figure 9:**
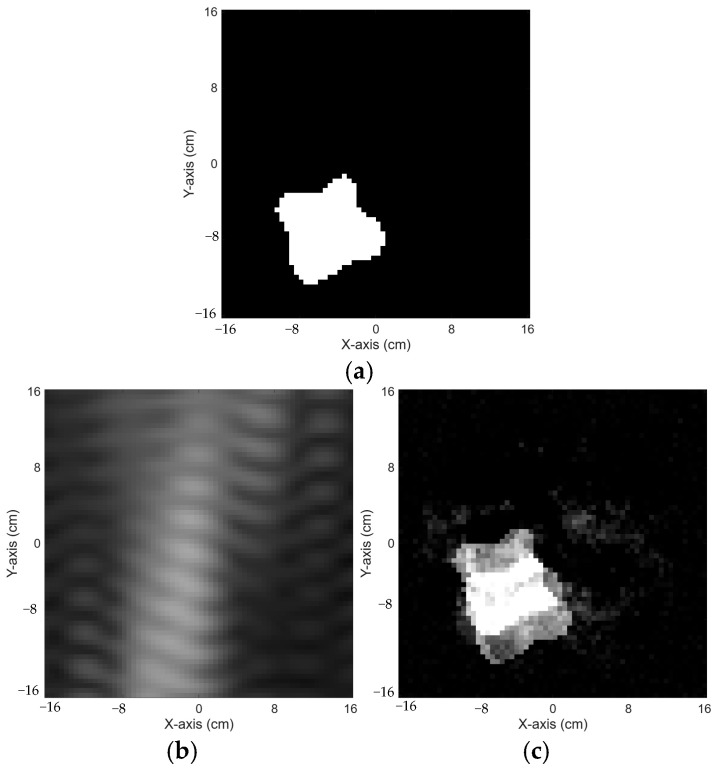

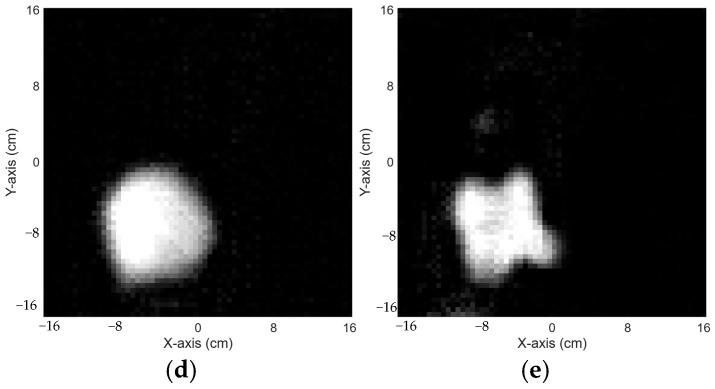
Comparison of conductor reconstruction results: (**a**) ground truth; (**b**) DSM-only reconstruction; (**c**) DCNN reconstruction at 3 GHz; (**d**) U-Net reconstruction at 2 GHz; and (**e**) U-Net reconstruction at 3 GHz.

Figure 9 shows that the DSM-only reconstruction fails to reliably identify the target location and contour, yielding an average NRMSE of 29.08%. In contrast, DCNN provides a recognizable reconstruction, while U-Net achieves the most accurate target contour with fewer reconstruction artifacts. At 3 GHz, DCNN achieves an average NRMSE of 14.11% and an average SSIM of 49.93%, whereas U-Net improves these values to 7.84% and 69.29%, respectively. At 2 GHz, U-Net yields an average NRMSE of 11.54% and an average SSIM of 58.12%. The improved performance at 3 GHz supports its selection as the operating frequency in the preceding experiments. Moreover, the consistent results obtained using the enlarged dataset further confirm the effectiveness of the proposed DSM–U-Net framework under the tested MoM-based simulation conditions.

All computations were performed on a workstation equipped with a 10th-generation Intel Core i7 processor, 64 GB of RAM, and an NVIDIA RTX 20-series GPU. Under this computational environment, network training required approximately 150 s and 2.5 GB of system RAM. The DSM reconstruction required approximately 250 s per sample, whereas the trained network completed the subsequent inference in less than 1 s. For comparison, under the same simulation and computational conditions, the genetic algorithm (GA) required approximately 5 h to complete 1000 generations and yielded an NRMSE of approximately 15% [20]. By contrast, the proposed DSM–U-Net framework achieved a lower average NRMSE of 7.84% with substantially reduced reconstruction time.

## 5. Conclusions

This paper proposes a framework combining the DSM and U-Net for reconstructing perfect conductors buried in slab media. As the buried conductor limits both the observation and reception angles, we first use the scattered field data calculated by the MOM to estimate a preliminary image using DSM. Simulation results demonstrate that the standalone DSM cannot effectively recover the conductor shape; it only provides an approximate location. Such deficiencies arise largely from the intensive nonlinearity intrinsic to TE waves and the constrained collection of comprehensive scattered signals emitted by the target. To overcome these limitations, this study introduces the U-Net architecture to refine the initial reconstruction results. The numerical results show that the U-Net refinement improves the reconstruction quality of the preliminary DSM images. Comparisons with the DSM-only and DCNN results further demonstrate the effectiveness of the proposed framework in terms of NRMSE and SSIM under the considered noise conditions. The demonstrated robustness is limited to variations in target shape, spatial position, and operating frequency within the adopted MoM-based forward-simulation model. A limitation of the present study is that all results are based on simulated data generated using the MoM, as no publicly available measured dataset is currently available for the considered TE-wave slab-medium configuration. Future work will therefore extend the present two-dimensional framework to finite three-dimensional targets, realistic lossy and heterogeneous media, practical antenna configurations, and experimental measurements while investigating advanced neural network architectures for higher-resolution and more computationally efficient reconstruction. These extensions will enable a more rigorous evaluation of the framework’s potential for practical applications, including nondestructive testing and subsurface sensing.

## Figures and Tables

**Table 1:** NRMSE performance for seven shapes by U-Net under 5% noise.

	Performance	NRMSE
Shape		
circle		6.77%
oval		5.75%
pea		5.41%
cross		8.46%
UFO		7.33%
four-petal		9.28%

**Table 2:** NRMSE performance for seven shapes by U-Net under training data with 5% noise and test data with 10% noise.

	Performance	NRMSE
Shape		
circle		8.15%
oval		6.29%
pea		9.61%
cross		14.51%
UFO		7.96%
four-petal		9.58%

## Data Availability

The original contributions presented in this study are included in the article. Further inquiries can be directed to the corresponding author.
